# Concomitant Pathologies and Their Impact on Parkinson Disease: A Narrative Overview of Current Evidence

**DOI:** 10.3390/ijms26072942

**Published:** 2025-03-24

**Authors:** Kurt A. Jellinger

**Affiliations:** Institute of Clinical Neurobiology, Alberichgasse 5/13, A-1150 Vienna, Austria; kurt.jellinger@univie.ac.at; Tel./Fax: +43-1-5266534

**Keywords:** Parkinson disease, co-pathologies, comorbidities, Alzheimer lesions, cerebrovascular, autoimmune diseases, diabetes mellitus, myasthenia gravis, COVID-19

## Abstract

Many clinico-pathological studies point to the presence of multiple comorbidities/co-pathologies in the course of Parkinson disease (PD). Lewy body pathology, the morphological hallmark of PD, rarely exists in isolation, but is usually associated with other concomitant pathologies, in particular Alzheimer disease-related changes (ADNC), cerebrovascular pathologies (macro- and microinfarcts, cerebral small vessel disease, cerebral amyloid angiopathy), TDP-43 pathology as well as multiple pathological combinations. These include cardiovascular disorders, metabolic syndrome, diabetes mellitus, autoimmune and rheumatic diseases, myasthenia gravis, Sjögren’s syndrome, restless leg syndrome or other rare disorders, like Fabry disease. A combination of PD and multiple sclerosis (MS) may be due to the immune function of LRRK2 and its interrelation with α-synuclein. COVID-19 and HIV posed considerable impacts on patients with PD. Epidemiological evidence points to a decreased risk for the majority of neoplasms, except melanoma and other skin cancers, while some tumors (breast, brain) are increased. On the other hand, a lower frequency of malignancies preceding early PD markers may argue for their protective effect on PD risk. Possible pathogenetic factors for the association between PD and cancer are discussed. The tremendous heterogeneity of concomitant pathologies and comorbidities observed across the PD spectrum is most likely caused by the complex interplay between genetic, pathogenic and other risk factors, and further research should provide increasing insight into their relationship with idiopathic PD (and other parkinsonian disorders) in order to find better diagnostic tools and probable disease-modifying therapies.

## 1. Introduction

Parkinson disease (PD), the second most common neurodegenerative disorder, is neuropathologically characterized by the widespread intraneuronal and neuritic deposition of abnormal phosphorylated α-synuclein-forming Lewy bodies (LBs) and Lewy neurites, the morphological hallmarks of PD and related LB disorders. They induce progressive degeneration not only of the dopaminergic nigrostriatal system, but of many other neuronal and extraneural systems; PD is thus a multisystem disorder. The site of origin of α-synuclein (αSyn) deposition is a matter of ongoing discussion, since it was suggested either to originate in the olfactory bulb and to progress upward to interconnected brain regions [1,2], or in the enteric system, propagating to the brainstem with caudo-rostral progression throughout the brain [3]. Similarly to other neurodegenerative disorders, brain co-pathologies have increasingly been recognized as a rule (>90%) and not an exception in PD, with PD patients exhibiting a mixture of different concomitant disorders [4,5,6]. They may be either coincidental or—more likely—have an influence on the disease development and/or its clinical presentation [7]. While microinfarcts, atherosclerosis, arteriolosclerosis, cerebral amyloid angiopathy (CAA) and tau pathology have an impact on disease progression and cognitive impairment (CI) [8,9], others, like β-amyloid (Aβ) and transactive response DNA binding protein of 43 kDa (TDP-43), do not seem to contribute to PD progression [10,11]. On the other hand, the co-occurrence and co-aggregation of TDP-43 with other pathogenic proteins may aggravate the disease [12].

Non-AD neurodegenerative co-pathologies and their combinations have been underestimated, but are frequent in reality [13]. The analysis of 140 autopsy-proven PD cases revealed AD in 38%, argyrophilic grains in 25%, cerebral white matter (WM) rarefication in 44% and CAA in 24% [14]. Another study described the combinations of nine pathologies related to progressive PD, including cerebrovascular pathologies (CVP) in 41–48%, CAA in 65%, TDP-43 and hippocampal sclerosis, with 31 combinations of the five major pathologies [5]. Alzheimer disease-related pathology (ADNC) and several CVPs were all independently related with progression of PD [9]. Neocortical and limbic αSyn pathology had the strongest association with dementia; tau pathology was moderate to severe in around one-third of cases and moderate to severe Aβ pathology was found in over a half. While cerebrovascular and TDP-43 pathologies did not generally contribute to dementia in PD, TDP-43 and CAA correlated with coexistent ADNC [11]. In a recent examination of the age-related prevalence of co-pathologies in 269 autopsied PD subjects, amyloid plaques were present in more than 40% in their 60s and in 85% of those in their 90s, CAA increased from 20% to 80% and neuritic Braak stages IV or greater affected 40% to 60% of subjects from their 70s to those in their 90s. White matter rarefication increased from 30% to 75%, microinfarcts from 25% to 50%, macroinfarcts from 10% to 20%, aging-related tau astrogliopathy (ARTAG) and argyrophilic grains between 20% and 40%, limbic-predominant age-related TDP-43 encephalopathy (LATE) from 20% to 50% and PSP from 5% to 10%, while coexisting Pick disease or motor neuron disease were not observed [15].

This article is intended to review the current knowledge about concomitant pathologies and their impact on PD. It will not include neuropsychiatric comorbidities, which have been reviewed recently [16,17,18]. The literature research strategies are described in the Supplementary Materials.

## 2. PD- and AD-Related Co-Pathology

Autopsy and experimental studies have confirmed the concomitant coexistence of Aβ and tau pathologies in PD brains [19], suggesting their synergistic interactions, which can contribute to altering the progression of the disease [20,21,22], although one study suggested that cortical Aβ and tau did not relate to cognition in PD patients without dementia (PDND) [23].

Several studies have demonstrated increasing evidence of tau pathology in PD brains [24,25,26]. Among 165 autopsy cases of PDND, the frequency of low or intermediate ADNC was 10.8%, while 26.7% of Parkinson disease dementia (PDD) showed intermediate to high ADNC [27], with high ADNC scores being significantly more frequent in PDD cases [28]. In 100 autopsy cases of PDND (mean age at death 77.9 ± 7 years), Braak tau stages ranged from 0 to 4 (mean 2.3), compared to 2–6 (mean 4.4 ± 0.4) in 110 PDD cases; the Thal Aβ phases in PDND had a mean of 1.8 ± 0.2 compared to 2–4 (mean 3.0 ± 0.2) and there was a CAA frequency in PDND of 2.4% versus 50% in PDD (all *p* < 0.001), while the degree of cerebrovascular lesions was 0.7 vs. 1.3 (*p* < 0.05) [29]. These and other postmortem data emphasize the importance of AD co-pathologies in the pathogenic continuum within the spectrum of age-related synucleinopathies. In a prion-type mouse model, αSyn enhanced the Aβ deposition, while pre-existing Aβ plaques exacerbated the spread and deposition of induced αSyn pathology; this process was associated with increased neuroinflammation. This revealed interplays between αSyn, Aβ and neuroinflammation in mice that recapitulated the pathology of PD and AD [30].

Genome-wide association studies (GWASs) have identified MAPT-encoding tau protein as being associated with an increased risk of sporadic PD [31,32], and tau aggregates have been described in familial PD as being linked to A53T αSyn mutation [33,34,35]. Tau and αSyn are both intrinsically unfolded proteins occurring in synapses that can adopt different conformations, including oligomers and abnormal intracellular aggregates. αSyn phosphorylation triggers tau pathology and induces widespread phosphorylated tau with a prion-like nature to spread to various brain areas in PD [36]. This supports the thesis that αSyn and tau form a deleterious process essential for the development and spreading of neurodegeneration in PD. Although αSyn and tau aggregates occur in the same PD brain, their aggregates are usually separated and only 5–8% of AT8 (phosphorylated tau) aggregates are co-localized with *p*-S129-αSyn in substantia nigrac neurons. These data suggest that there is no direct relationship between tau and αSyn aggregates and that the initiation of nigrostriatal dopaminergic degeneration is independent of αSyn aggregation and could be tau-mediated [4]. At later stages of PD, tau aggregates are rarely detected in the nigrostriatal system and may disappear, concomitant with dopaminergic neuronal death. Tau aggregates in the nigrostriatal system have also been observed in parkinsonism without LB pathology, suggesting that tau accumulation may be upstream of αSyn aggregation and precede the occurrence of LBs in the substantia nigra (SN) and in stages in which tyrosine hydroxylase-positive neurons are already reduced [37].

## 3. PD and TDP-43 Pathology

TDP-43 inclusions are the morphological hallmark of amyotrophic lateral sclerosis (ALS) and a subset of frontotemporal lobe degeneration (FTLD-TDP), but TDP-43 inclusion has been identified in age-related neurodegenerative disorders, referred to as limbic-predominant age-related TDP-43 encephalopathy (LATE) [38]. TDP-43 pathology initially involves the amygdala, then progresses via limbic areas to the neocortex [39,40]. The accumulation of a novel site-directed caspase cleavage antibody (TDP caspase cleavage product antibody/TDPccp) has been observed in Lewy and Hirano bodies in all cases of PD (and dementia with Lewy bodies/DLB). The co-localization of TDPccp with an antibody to αSyn, which serves as a general marker for LBs, was evident in PD with and without dementia (and DLB). The TDPccp antibody detected a greater number of LBs in PD (and DLB) compared to the αSyn antibody [41]. The synergistic association of TDP-43 and αSyn promoted the aggregation of both proteins and enhanced neurodegeneration in vivo and in vitro [42,43,44,45]. αSyn aggregates also have a synergistic effect on tau and TDP-43 aggregation [46]. Although the precise mechanisms behind this are unclear, the accumulation of DTP-43 and mutant αSyn synergistically induce dopaminergic neurodegeneration [45]. PARKIN and PINK1 are regulators of mitophagy, an autophagic pathway for the selective elimination of dysfunctional mitochondria. PARKIN depletion has been associated with recessive early onset PD caused by loss-of-function mutations in the PARK2 gene, while in sporadic PD, the activity and abundance of this protein can be compromised by stress-related modifications. PARKIN depletion is not limited to PD, but is also observed in other neurodegenerative diseases, especially those characterized by TDP-43 proteinopathies such as LATE and ALS [47]. Moreover, TDP-43 pathology is present in most post-encephalitic parkinsonian brains [48]. It also occurs in Perry disease, a hereditary neurodegenerative disorder with autosomal dominant inheritance that is characterized by parkinsonism, psychiatric symptoms, unexpected weight loss and cerebral hypoventilation. It is caused by the dynactin I gene (DCTN1), which encodes dynactin subunit p150 on chromosome 2p. The dynactin complex is a motor protein associated with axonal transport. The neuropathology of Perry disease reveals severe degeneration in SN and TDP-43 inclusions in the brainstem and basal ganglia [49].

ARTAG that is associated with LATE and cerebrovascular pathologies but not with ADNC [50] also coexists with up to 80% of PD, but has no association with either final MMSE or motor scores [15].

Argyrophilic grain disease (AGD), an age-related 4R-tauopathy affecting the medial temporal lobe, although being a rare co-pathology, may induce cognitive decline and psychiatric symptoms in PD [51,52].

## 4. Cerebrovascular Disease

Cerebrovascular disease, including macro- and microinfarts, lacunes, hemorrhages, WM lesions and CAA are frequently present in PD brains [53,54,55,56,57]. Cerebral small vessel disease (CSVD) has a causal role in parkinsonism [58] and the severity of PD [59,60]. Neurovascular coupling alterations in the striato-thalamo-cortical circuit can be driven by dopaminergic systems [61]. The increased prevalence of CSVD has been seen in PD (44%) compared to 32.8% in controls. The severity of cerebrovascular lesions in PD ranged from mild to moderate to severe (26.8%, 19.6% and 6.8%), versus 20.9%, 6.5% and 5.4% in controls [53]. The extent and severity of CSVD had a considerable impact on cognitive impairment (CI) in PD. A meta-analysis showed its effect mainly on executive and overall cognitive functions [8,62], whereas others found no correlations between CSVD pathology and CI [59,63]. Lacunes in the basal ganglia and WM are associated with cognitive decline and may accelerate the course of PD [64,65], as are microbleeds, and both of which are associated with hypertension [66]. Disorders of the blood–brain barrier (BBB) related to microvascular changes have been observed in early PD [67].

The vascular pathology in PD ranges from early pericyte activation and leakage of the BBB to final vascular rarefication [68], lacunes and WM hyperintensities as markers of small vessel disease [56].

Atherosclerosis and macroscopic and microscopic infarcts interact with LB pathology to increase the severity of parkinsonian symptoms [69]. Comorbid WM lesions, lacunes and microbleeds due to CSVD are associated with the worsening of motor and cognitive impairment, probably independent of the degree of nigrostriatal dopaminergic denervation in PD [59,70,71], which may be due to disturbance of functional brain networks [72]. PD is related to an elevated risk of cardio-cerebrovascular diseases, including cardioembolic stroke [73]. Cardiovascular diseases and PD share pathological processes, particularly inflammation, insulin resistance, lipid metabolism and oxidative stress (OS), while smoking and low-density cholesterol appear to have opposite associations with cardiovascular disease and PD [74].

## 5. Prevalence of Other Comorbidities

Studies in large cohorts of PD patients have confirmed the high prevalence of somatic comorbidities; 92.9% had at least one somatic comorbidity, with the most prevalent being hypertensive diseases (67.71%), ischemic heart disease (42.74%), diseases of the musculoskeletal system and connective tissue (39.21%) and metabolic syndrome (33.24%). These comorbidities increase with PD progression and their prevalence increases with age [75]. The impact of many of these comorbidities was the focus of expert panels [76], whereas others have discussed the association of vascular risk scores with global cognition in PD [77]. Among veterans with PD, the most frequent comorbidities were coronary heart disease, arthritis, chronic low back pain, congestive heart failure, diabetes and stroke [78]. Chronic pain was identified in 19.3% of males and 22.8% of females with PD [79]. The prevalence of psychiatric comorbidity in patients with PD was 77%, with most having multiple co-occurring psychiatric symptoms [80], while in India, two-thirds of PD patients had associated psychiatric comorbidities [81]. For comparison, in a large cohort of patients with progressive supranuclear palsy (PSP), the most frequent comorbidities were diabetes and cardiovascular diseases [82]. The top five comorbid diseases as contributory causes of death in PD in the Japanese population were cerebrovascular diseases (4%), dementia (3.8%), diabetes mellitus (3.6%), malignant neoplasm (2.5%) and heart disease (2.3%) [83]. The co-occurrence of PD with retinitis pigmentosa may be related to mutations in cyclic nucleotide gated channel subunit alpha 1 (CNGA1) and SNCAIP genes [84].

## 6. Diabetes Mellitus and Metabolic Syndrome

There is an emerging body of evidence about the links between PD and type 2 diabetes mellitus (T2DM) that share common pathways [85,86]. Significantly elevated rates of PD following T2DM were greater in those with complicated T2DM [87]. According to a recent meta-analysis encompassing a total of 39,209,316 participants, the relative risk concerning the association between diabetes mellitus and PD yielded a value of 1.22 (95%CI 1.08–1.37) [88], and for prediabetes, 1.09 (95%CI 1.02–1.16), which may not have been significantly influenced by the country of the study or the age and sex of participants [89]. Both PD and T2DM share misregulated processes, including mitochondrial dysfunction, OS, inflammation, protein aggregation, altered proteostasis and dysregulation of glucose metabolism. Hyperglycemia, a T2DM hallmark, can lead to increased methylglyoxal production, which is responsible for protein glycation. Methylglyoxal plasma levels and αSyn glycation are significantly elevated in T2DM patients, which suggests a common molecular mechanism [90], while glycemic variability (fluctuating blood glucose levels) causes OS and endothelial damage, which are also active in PD [91]. Other shared disease mechanisms of insulin dysregulation are mitochondrial dysfunction, neuroinflammation, the aggregation of amyloid, altered synaptic plasticity [85] and an interplay between OS, reactive oxygen species and ferroptosis in PD and T2DM [92]. Furthermore, several amino and nucleic acids and fatty acid metabolism are associated with T2DM only in PD patients, suggesting a possible link between both disorders [93]. MicroRNAs that play an important role in cell differentiation, the regulation of the cell cycle and apoptosis are involved in both PD and T2DM, since they regulate the insulin pathway and glucose absorption, and can also regulate PD-related genes [94]. Genetic profiles of T2DM-PD patients show similarities, and potential risk factors include insulin resistance and dysbiosis of the gut–brain microbiome [95,96,97]. T2DM is independently associated with severe CI in PD [98].

Type 1 diabetes mellitus (T1DM) is associated with a reduced genetic susceptibility to PD [99]. These findings were replicated with another independent GWAS data set, providing evidence that T1DM may have a protective effect on PD risk [100]. Another inverse-variance weighted Mendelian randomization of the large datasets of genomes involving European populations revealed a reduced genetic susceptibility to PD, which means that T1DM has a reduced genetic susceptibility to PD, but further studies are warranted to elucidate the underlying mechanisms and provide a more comprehensive understanding of this relationship [99].

Inconsistent results regarding the association between the metabolic syndrome (MetS) and PD have been reported. This association is especially due to elevated serum triglycerides and plasma fasting glucose concentrations. After exclusion and further adjustment for other components of the MetS, an increased PD risk was observed in overweight individuals, indicating that a high BMI (body mass index) seems to be related to an increased PD risk [101]. MetS has an effect on non-motor symptoms in PD [102] and contributes to CI and its severity in PD [103].

High blood pressure, low high-density lipoprotein cholesterol, and high fasting blood glucose levels increased the PD incidence by 1.34, 1.31 and 1.20 times, respectively [104]. A nationwide cohort study confirmed that persistent and incident MetS may be a risk factor for incident PD [105]. MetS and PD share multiple pathophysiological processes, including insulin resistance, OS and chronic inflammation, although the specific mechanisms remain unclear [106]. However, a recent cross-sectional study showed that MetS does not significantly affect the risk of PD (OR: 1.01), while hypertension and diabetes mellitus significantly increase it [107].

## 7. Autoimmune Diseases (Table 1)

An increasing amount of epidemiological evidence points to the potential relationship between PD and various autoimmune disorders (AiDs); the 33% overall presence of PD among patients with AiDs being significantly higher than in the general population [108]. An increased risk of PD was associated with bullous pemphigoid (OR = 2.67), ulcerative colitis (OR = 1.31), inflammatory bowel disease (OR = 1.30), Sjögren’s syndrome (SS) (OR = 1.61), Graves’ disease (OR = 1.45) and Crohn’s disease (OR = 1.30). There appears to be no significant association with systemic lupus erythematosus (SLE) (OR = 0.82), multiple sclerosis (MS) (OR = 2.02), rheumatoid arthritis (RA) (OR = 0.79) and celiac disease (OR = 1.16). This study supports the strong link between AiDs and PD. However, since most of the included studies were retrospective, larger prospective cohort studies are needed to explore the interaction between both disorders [109]. A meta-analysis showed no significant association between rheumatic arthritis, gout, SLE and polymyalgia rheumatica and PD, while ankylosing spondylitis, SS and Behçet’s syndrome may have increased PD risk [110]. There was no significant association between autoimmune hepatitis and subsequent PD, while the standardized rate ratio of PD following hepatitis C was 1.51 [111].

**Table 1:** Autoimmune diseases associated with Parkinson disease.

Type of Disorder	References
Allergic rhinitis	[112]
Autoimmune hepatitis	[111]
Bullous pemphigoid	[109]
Celiac disease	[109]
Crohn’s disease	[109,113]
Graves’ disease	[109]
Hashimoto thyroiditis	[114]
Inflammatory bowel disease/syndrome	[109,115,116,117,118,119,120,121,122,123,124,125,126]
Multiple sclerosis	[109,127,128,129,130,131,132,133,134]
Myasthenia gravis	[135,136,137,138,139,140,141,142,143]
Osteoarthritis	[144,145]
Polymyalgia rheumatica	[110]
Rheumatoid arthritis	[109,110]
Sjögren’s syndrome	[111,146,147,148,149,150,151,152,153,154,155]
Systemic lupus erythematosus	[109]
Ulcerative colitis	[109]

In systemic AiDs such as SLE, antiphospholipid syndrome and SS, parkinsonism is attributed to the action of humoral and cellular immunity in specific brain regions such as the basal ganglia [156]. Recent GWASs and pathway analyses identified many novel loci with an overlap between PD and AiDs, identifying a common genetic pathway between these disorders [157]. However, a bidirectional Mendelian randomization (MR) analysis of GWAS suggested that the genetically predicted higher liability of PD was causally associated with a decreased risk of irritable bowel syndrome (OR = 0.98; *p* = 0.032). On the contrary, a potential positive correlation between genetically determined PD and the incidence of T1DM (OR = 1.10; *p* = 0.01) was detected. MR analysis therefore did not provide evidence to support the causal relationship of genetic predisposition to AiDs with PD susceptibility [158]. On the other hand, patients with allergic rhinitis had a higher risk of PD [112].

### 7.1. Sjögren’s Syndrome

Previous studies have reported an association between SS, a multisystem autoimmune disorder, and an increased risk of PD [146]. A nationwide population-wide cohort study in Taiwan found the risk of PD to be 1.37 times greater in AiDs patients than in controls, with RA and SS being associated with a significantly higher risk of PD [159]. Another meta-analysis supported that people with SS are at higher risk of PD and dementia [147]. A nationwide case–control study also proposed that SS is significantly associated with an increased risk of PD [148], with SS being an independent risk factor for PD [149]. Their connection was explained by the aquaporin link [150], which is involved in SS [151].

A transcriptome analysis revealed the presence of overlapping mitochondria-related genes and mitochondrial DNA damage in patients with primary Sjögren’s syndrome (pSS) and PD. Reactive oxygen species, the senescence marker p53, and the inflammatory markers CD45 and Bcl-2 were found to be regionally disturbed in the labial minor salivary gland of pSS patients. A weighted Gene Co-expression Network Analysis identified the STING (stimulator of interferon genes) pathway as the central mitochondria-related pathway which is closely associated with the immune system. Single cell analysis, immunohistochemistry staining and quantitative real-time PCR confirmed the activation of the STING pathway. Subsequent bioinformatic analysis revealed the proportion of infiltrating immune cells in the STING-high and -low group of pSS and PD. This study demonstrated the association of the STING pathway with innate and adaptive immune cells in the microenvironment of PD and pSS. It uncovered a central pathway between mitochondrial dysfunction and the microenvironment in PD and pSS, potentially offering valuable insight into the relationship between the two conditions [152].

However, a recent bidirectional MR analysis using a GWAS of European ancestry gave no evidence of a significant causal effect of SS on PD risk, and similarly, no evidence supported the causal effects of PD on SS, but further studies to clarify the mechanisms of a probable causal effect between the two disorders are necessary [153]. The other weighted median, inverse variance weighted, MR-pleiotropy residual sum and outlier methods demonstrated a significant association between predicted SS and a reduced risk of PD, while a sensitivity analysis confirmed the robustness of the causal relationship between PD and SS; reverse MR analysis did not support any causal effects of PD on SS. This MR study supported a potential association between SS and a reduced risk of PD [154]. Another MR analysis of GWAS data also revealed no significant association between SS and PD (OR = 1.00; *p* = 0.95), which contradicts numerous existing observational reports [155].

### 7.2. Rheumatoid Arthritis

Rheumatological manifestations are common in PD [160] and coexistent osteoarthritis seems to additively increase the risk of mortality [144]. A population-based longitudinal follow-up study suggested that osteoarthritis was linked to an increased risk of PD [145].

PD was found more frequently in elderly patients with RA compared to controls [161], and the risk of PD was 1.37 times greater in autoimmune rheumatoid diseases [159]. A comparison of DNA methylation linked RA and PD, a link which was larger than between RA cases and controls. There was a total of 337 gene pairs with large changes shared between RA and PD, suggesting possible pathobiological mechanisms between RA and PD [162]. On the other hand, RA was associated with a 30–50% lower risk of developing PD [163]. This reduction was independent of treatment with antirheumatoid drugs, which appeared to further reduce this risk [164]. An MR analysis based on large GWASs from RA and PD identified a significant negative correlation between both (*p* = 0.0033), while an increase in RA risk was associated with a lower risk of PD. This supported a protective role of RA on PD [165], which was confirmed by other MR analyses [166], and also for European populations (*p* = 0.004). A multivariable MR analysis demonstrated that only immunosuppressants were related to a decreased risk of PD, but not glucocorticoids or NSAIDs (non-steroidal anti-inflammatory drugs) [167].

### 7.3. Myasthenia Gravis

Comorbidity between PD and myasthenia gravis (MG), an AiD caused by antibodies against the neuromuscular junction, is rare, with positive anti-muscle specific kinase reported in only one case [135], while others reported 15 cases of PD and MG [136]. In the largest reported series of concurrent MG and PD, this concurrence was more common than expected (2.85%). Either MG or PD may appear first [137]. The male sex significantly prevailed, as well as the presence of multiple comorbidities in patients with MG associated with PD. In terms of the clinical course, MG was benign, as most cases remained stable (66.7%) [138]. On the other hand, PD patients may show dropped head syndrome characterized by severe forward flexion of the cervical spine due to an imbalance in neck muscle tone, which may be indistinguishable from antecollis in MG and requires a tailored treatment approach [139,140,141]. The underlying mechanisms for the coexistence of PD and MG remain unknown, but an imbalance between the neurotransmitters dopamine and acetylcholine and the immune system is likely to play an important role in the pathogenesis [142]. The treatment of PD and MG must rely on monitoring new symptoms and warrants future research [143].

### 7.4. Inflammatory Bowel Disease

PD and inflammatory bowel disease (IBD) are chronic disorders affecting the CNS and gastrointestinal tract, respectively. Recent research suggests a bidirectional relationship between neurodegeneration in PD and intestinal changes in IBD. PD patients may experience gastrointestinal dysfunction over a decade before motor symptom onset, and IBD may increase the risk of developing PD [168], which is significantly higher than in controls [169]. Crohn’s disease (CD) shows a 27% increased risk of PD [170]. This was confirmed by a meta-analysis of 14 studies, while IBD medication was suggested to have a protective role against PD development [171].

Despite the “gut–brain axis” concept, the underlying pathomechanisms of this potential association remain unclear. There is robust evidence for a genetic link between PD and IBD, indicating both synergistic and antagonistic pleiotropy [115]. Gene marker analysis identified five important genes, among which BTK and NCF2 allowed the discrimination of both IBD and PD [116]. Among the other genetic loci shared between both disorders is LRRK2 (leucine-rich repeat kinase 2), initially identified as a causal gene in PD, and recently also implicated in CD, suggesting a link between these seemingly unrelated diseases which share genetic susceptibility [117]. LRRK2 is detected in inflamed colon tissue from IBD and in peripheral cells from sporadic PD, indicating that both share overlapping phenotypes, particularly in terms of LRRK2 in the context of the immune system [118].

The analysis of LRRK2 missense variants revealed a significant association of the G2019S and N2081D variants with IBD-PD, in addition to several other variants as potential contributors to increased or decreased IBD-PD risk. There is prominent overlap between the enriched pathways in the known IBD-PD and candidate IBD-PD gene sets. Additionally, significantly enriched pathways unique to the IBD-PD gene sets were detected, and 14 final candidate IBD-PD genes were prioritized by biological relatedness methods. The estimated protein–protein interaction analyses indicated the involvement of genes related to immunity, inflammation and autophagy in IBD-PD. An additional PheWAS (phenome-wide association study) provided support for the association of candidate genes with IBD and PD. This study confirmed and uncovered new LRRK2 associations in IBD-PD. The identification of novel inflammation and autophagy-related genes supports and expands previous findings related to IBD-PD pathogenesis [119]. PARK7 has also an immunomodulatory role in IBD-related inflammation [120].

A meta-analysis of the temporal relationship revealed that the incidence of IBD was significantly increased before and after PD diagnosis, suggesting that IBS may moderately increase PD risk regardless of sex, especially in people over age 65 [121]. Conversely, bidirectional two-sample MR studies using GWAS summary statistics of IBD and PD provided no evidence for their causal association [122]. A prospective cohort study of 468,556 UK biobank participants over an average follow-up period of 13.9 years also found no significant association between IBD and PD [123]. Another two-sample MR study from available GWASs gave no evidence for an association between IBD and PD (OR = 0.98), suggesting that neither IBD nor its subtypes (CD and ulcerative colitis) causally affect PD in the European population [124]. This was confirmed by another MR analysis of GWASs of European descent cases [113]. On the other hand, the existence of pathogenic αSyn in both the gut and the brain reinforces the potential role of the enteric nervous system as a contributing factor in PD etiology. A DSS-based rat model of gut inflammation demonstrated the appearance of phosphorylated αSyn inclusions in both Auerbach’s and Meissner’s plaques (gut), increased αSyn expression in the ventral mesencephalon and the degeneration of nigral dopaminergic neurons, the classical hallmarks of PD. These findings emphasize the significance of peripheral inflammation and the gut–brain axis in inducing αSyn aggregation and transport to the SN, resulting in neurodegeneration [125]. A systemic review and meta-analysis of longitudinal studies verified the two-directional association between the gut–brain axis, specifically between IBD and neurodegenerative disorders, including PD [126].

### 7.5. Hashimoto Thyroiditis

Hypo- and hyperthyroidism (Hashimoto and Graves’ disease) not only increase the risk of PD but also show some clinical signs of PD. Hypothyroidism is associated with anemia, hypercholesterolemia and altered cerebral blood flow, which are associated with PD pathology. Thyroid stimulating hormone (TSH) stimulates dopamine release; PD is associated with the impaired regulation of TSH and thyroid hormone [114]. Autoimmune thyroiditis associated with parkinsonism may resemble PD [172]. On the other hand, population-based studies did not identify an association between gout and risk of PD [173,174], and a Mediterranean population-based study showed PD-protective gout in both men and women after age 75 years [175].

## 8. PD and Multiple Sclerosis

The association between MS and parkinsonism is rarely reported [128,176,177] and there is still an open debate regarding whether MS lesions can cause parkinsonian symptoms or whether the coexistence of both diseases in the same patient is accidental. A literature review reported 42 cases of co-occurrence of parkinsonism and MS, including a case of concomitant MS and PD diagnosed on both clinico-radiological and cerebrospinal fluid αSyn findings [176]. However, in the majority of cases, parkinsonian symptoms developed during the course of MS [178] and reacted to methylprednisolone but not to levodopa [179]. Only single cases of both circumstances have been reported, in which the parkinsonian syndrome developed first [127,129], or young-onset PD and a heterogenous point mutation in PARKIN was associated with the later development of primary progressive MS [130]. Another PD occurred in a patient with glucocerebrosidase gene mutation and MS [180]. The frequency of PD associated with MS was suggested to be 1:12.5 million [128]. A possible relationship between MS and LRRK2 PD has also been suggested. However, neuropathological studies of homozygous LRRK2 carriers with PD are rare and there are no systemic reports on the clinical features in those cases. MS preceded PD in 1.4% of carriers with LRRK2 G2019S variants, and in none with idiopathic PD (*p* = 0.03). One case with MS and PD was a LRRK2 G2019S homozygous carrier, and neuropathology showed the degeneration of SN compacta without LB deposition, as well as multiple WM lesions consistent with MS. The co-occurrence of MS and LRRK2 PD, although rare, further supports an important role for immune function in LRRK2 PD and that MS may be an expression of the LRRK2 G2019S variant that includes both MS and PD, with MS predating features characteristic of PD. Neuropathology suggests that MS occurs independent of αSyn deposition and that LB pathology may be absent in homozygotic LRRK2 carriers [131]. A subset of patients with LRRK2 mutations present with a clinical phenotype indistinguishable from iPD but lacking LBs. This suggests that LRRK2-mediated PD may occur independently of αSyn aggregation [132]. Further studies examining the interaction between the two proteins are warranted in order to elucidate their role in the co-occurrence of MS and PD. On the other hand, αSyn immunoreactivity in neurons, microglia and oligodendrocytes in MS brains may be related to neuroinflammation [133]. Moreover, PD and MS share commonalities in terms of iron accumulation in the SN, which may be due to protein–protein interaction networks related to metal homeostasis [134].

## 9. PD and Other Pathologies

PD can be associated with many other diseases and multiple pathologies can be observed in the PD brain at postmortem examination: A growing body of evidence suggests that concomitant pathologies can contribute to changes in the clinical symptomatology and to cognitive decline. It is assumed that at least some of the concomitant pathologies have a synergistic relationship with PD and research is ongoing to resolve the pathomechanisms underpinning this to enable the design of disease modifying treatments.

*Amyotrophic lateral sclerosis*: The coexistence of ALS with PD, although rather uncommon, is found to a greater degree than one would expect by chance. Parkinsonian features have been reported in up to 30% of ALS patients and LBs have been reported in a certain number of ALS cases [181]. This unique co-occurrence has been referred to as Brait–Fahn–Schwartz disease [182]. The causes and pathomechanisms of the selected neurodegeneration in both disorders are not fully understood, but their coexistence suggests that they could share common biomechanisms [183]. An older multicenter study demonstrated that there is a strong association between PD, ALS and the gene encoding angiogenin (ANG) variants, which may be a genetic link between both disorders [184]. Biochemical, molecular genetics and animal model studies have suggested that malfunctioning mitochondria might contribute to neuronal death in both PD and ALS [185], which was confirmed by later studies [186]. Others have discussed serotonergic dysfunction related to the common lesioning of the raphe nucleus [187] or peripheral immune activation as shared pathogenic pathways [188,189]. Genetic studies have supported the role of MMP-9 (matrix metalloproteinase-9) polymorphism in the risk of PD and ALS [190], whereas modern GWASs have identified eleven genetic risk loci shared among PD, ALS and AD. This supports lysosomal dysfunction, OS and DNA damage response as being transdiagnostic processes underlying the pathogenesis of these disorders [191]. A meta-analysis of de novo MR studies indicated that genetic predicted levels of various physical activities were related to a raised risk of ALS, but not causally with PD [192]. The description of PD-ALS clinical complex supports the hypothesis of there being common neuropathological pathways related to the frequent co-occurrence of αSyn and tau aggregation in such brains, suggesting the synergistic interaction of these (and other) proteins in determining the neurodegenerative process. A recent case report in a 54-year-old Italian woman with iPD later complicated by ALS carrying a novel MAPT variant (Pro494Leu) supported the hypothesis of a genetic dysfunction as the basis of multiple neurodegenerative disorders [193]. The Parkinson-dementia and ALS association complex of Guam [194,195] is not a matter covered in this review.

*Fabry disease*: PD with lower α-galactosidase A (α-GAL) enzymatic activity can be associated with Fabry disease (FD), an X-linked lysosomal storage disease caused by α-GAL A deficiency. The prevalence of PD in FD is 1.3%, and 3% in persons aged over 50 years. PD in a late-onset phenotype of FD presents high cerebrovascular burden and a weak response to levodopa [196]. However, the few studies on this topic are heterogeneous, and the results are controversial. A hitherto unknown variant in the GAL gene was detected in PD patients in one large study, whereas decreased α-GAL activity was found in PD subjects in other studies, but without confirmation by lyso-Gb3 (globotriaosylsphingosine) assessment or GLA gene sequencing. A recent series of studies regarding PD patients from Southern Italy found no association between PD and FD, but definite conclusions were not possible due to the limited sample size [197].

*Fragile X syndrome*: Parkinsonism has been reported in fragile X-associated tremor/ataxia syndrome (FXTAS), a late-onset neurodegenerative disorder associated with premutation alleles (55–200 CGG repeats) of the fragile X mental retardation 1 (FMR1) gene. FXTAS is characterized by ubiquitin-positive inclusions in neurons and astroglia and by cerebellar tremor and ataxia. Parkinsonism in most FXTAS patients lacks rigidity and characteristic rest tremors, but parkinsonism in some is indistinguishable from PD. Therefore, FMR1 was proposed to be recognized as one of the exceptional genetic causes of parkinsonism with presynaptic dopaminergic loss and LBs [198].

*Progressive supranuclear palsy*: The pathological changes in PD and PSP have been reported to coexist. In the Arizona Study of Aging and Neurodegenerative Disorders between 1997 and 2014, 12 out of 125 cases with autopsy-confirmed PD had coexisting PSP pathology (9.6%). This observation suggests that coexisting PSP pathology may contribute to the clinical heterogeneity in PD and be a potential confounder in diagnosis [199].

*PD and restless leg syndrome* (RLS): The association between RLS and PD remains controversial, with epidemiological and descriptive evidence suggesting some potential overlap, while mechanistic/genetic studies suggest the negative independence of these conditions [200]. PLS prevalence estimates range from 0% to 52% in PD, rising from 4.6% at baseline evaluation to 16.3% after 4 years. PD patients with RLS have a higher age at PD onset, with more preserved dopaminergic pathways and cardiovascular disturbance [201]. The prevalence of RLS in Europe and North America was reported to be between 5% and 12%; in Asia, it was less than 4% [202]. A more recent meta-analysis reported a pooled RLS prevalence in PD among various populations of 14%, with prevalence in Asia (12%) being slightly lower than outside Asia (16%). While some studies stated that the prevalence of RLS in their PD patients did not significantly differ from the general population [203,204,205], a recent meta-analysis with a pooled RLS prevalence of 20% exceeded that in the general population, suggesting the existence of a relationship between the two disorders, and suggesting that neurotransmitter systems other than the dopaminergic one are involved in PD-RLS etiology [206]. The RLS frequency in de novo PD patients was higher than that in the general population [207]. RLS was more frequent in women (68% or 69% vs. 32%; *p* < 0.001) [208,209]. This was confirmed by others [210,211]. RLS may be an early feature of PD [212,213,214], whereas it may also delay the course of PD [215]. RLS and PD have been suggested to be two disorders that can coexist, whether or not they share a common pathophysiology [216]. RLS comorbid with PD was regarded as a heterogenous condition, since it may represent a prodromal feature of the neurodegenerative disease as an epiphenomenon of somato-sensory small fiber pathology [217]. However, FMR studies did not demonstrate any causal effect of PD on RLS, and linkage disequilibrium score regression analysis demonstrated a lack of genetic correlation between RLS and PD. This indicated no evidence for a causal relationship or genetic correlation between RLS and PD. The association observed in epidemiological studies could be attributed, in part, to confounding or nongenetic determinants [218].

## 10. PD and COVID-19

The risk of COVID-19 and PD was uncertain, but PD per se was an independent risk factor for hospitalization and related death because of a more severe health status and a high infection risk [219]. PD patients who contracted COVID-19 had a decline in motor functions, but the prevalence and prognosis of mortality seemed comparable to those without PD [220]. A meta-analysis showed that the pooled prevalence of COVID-19 infection in PD cases was 5%, not including hospitalization and mortality rates, which were 49% and 12%, respectively [221]. In a series of 104 PD patients with a confirmed COVID-19 infection, there were no differences in disease duration between patients with and without comorbidities. COVID-19 was less common in older patients (>50 years) with a longer PD duration. Amantadine treatment did not affect the risk or the severity of COVID-19 infection [222]. A multicenter UK-based study on PD patients hospitalized with COVID-19 showed that they needed increased respiratory support and required an increase in their care level post-discharge [223]. A few cases of parkinsonism linked to COVID-19 infection have been reported so far, raising the possibility of a post-viral parkinsonian syndrome. A comprehensive literature review confirmed the appearance of parkinsonism during or immediately after COVID-19 infections to be a rare event. Different mechanisms may play a role, including vascular damage, neuroinflammation, or the impact of SARS-CoV-2 on αSyn [224], but long-term observational studies, to the best of our knowledge, are not available. A recent review of 26 cases of post-COVID parkinsonism reported typical parkinsonian motor syndrome (*n* = 12), parkinsonism with postural instability and gait disorder (*n* = 3), and encephalopathy with parkinsonism (*n* = 10). Among the heterogenous clinical diagnoses, established PD was described in three cases, but there was no demonstration of a causative role of COVID-19, which could have been coincidental in several cases, whereas the others were acquired or unclassified parkinsonism [225].

*AIDS*: Parkinsonism with HIV infection is well known [226] and AIDS patients are susceptible to extrapyramidal symptoms [227]. HIV-associated PD has been shown to be similar to iPD, with some features suggesting an HIV-induced functional adaptation of dopaminergic neurons that might counteract the PD-related neuronal loss. PD-HIV cases had a lower Unified Parkinson’s Disease Rating Scale motor score and Hoehn and Yahr stage; concurrent HIV infection did not compromise the outcome of iPD [228]. People living with HIV and PD presented symptoms at a younger age, progressed slower to a severe stage and responded well to dopaminergic therapy. There were no statistically significant differences in the clinical phenotype, impulse control disorders and levodopa equivalent daily dose between the two groups, but PD-HIV had a higher frequency of dopamine dysregulation syndrome [229]. Alterations in brain signal oscillations in older patients with HIV infection and PD were similar. HIV patients mainly showed abnormal cortical amplitudes of low frequency fluctuations (ALFF) with reduced prefrontal amplitude and enhanced sensorimotor and inferior temporal amplitudes. Frontal hypoactivation overlapped for HIV and PD groups. These changes were associated with cognitive and motor dysfunction due to the disruption of neurofunctional frequency dynamics in subcortical–cortical circuits [230]. Episodic memory defects occurred in people with HIV and those with PD due to the similar impairment of frontal (precentral, orbital, superior, middle, inferior, supplemental motor) and limbic (hippocampus, thalamus) areas. Diminished learning in PD but not HIV was related to the smaller frontal superior volume, suggesting different pathogenic mechanisms for the neuronal correlates of memory deficits in HIV and PD [231].

*Creutzfeldt–Jakob disease*: The co-occurrence of PD with Creutzfeldt–Jakob disease (CJD) is rare [232], with only a single autopsy-confirmed case of overlapping disorders [233,234], while parkinsonism is a well-known clinical manifestation of CJD due to the affection of the dopaminergic and striatal outflow neurons or the loss of cholinergic interneurons in the striatum [235]. Recent studies suggest that PD can be differentiated based on where in the body the misfolded αSyn originates (either the brain or gut), similar to patients developing CJD or variant (v)CJD, human and animal model data indicate that αSyn and prion protein PrP misfolding originate in the gut in body-first PD and vCJD [236].

## 11. PD and Cancer Risk/Incidence

A link between PD and cancer has been supported by many epidemiological studies, most of which show that PD patients have a lower risk of developing most cancers than the general population, while increasing evidence points to a positive correlation with specific types of cancer, like melanoma [237]. For some tumor types, the risk was comparable to that in the general population [238,239]. However, the mechanisms underlying these epidemiological observations are not known.

Matched cohort analysis among 22,000 participants during a median follow-up of 5.2 years reported less cancer development in PD patients vs. controls (11.0% vs. 14.0%). Relative risk (RR) was present for smoking-related cancers such as lung (RR 0.32), colorectal (RR 0.54) and bladder (RR 0.68), as well as for most non-smoking-related ones, like prostate cancer (RR 0.74). In contrast, PD patients showed a significantly increased risk for melanoma (RR 6.15), a finding consistent with prior studies [240]. A meta-analysis of 29 studies showed a significantly reduced risk of cancer in PD patients compared to controls (31%) after excluding skin tumors (27%), with both smoking- and non-smoking-related cancer rates being significantly lower among PD patients [241]. In a Danish register, the overall cancer risk in PD patients was decreased, but there was an increased risk of malignant melanomas, non-melanoma skin cancer and breast cancer. However, among patients with early onset PD, the cancer risk was comparable to that of the general population [238]. The increased prevalence of malignant melanoma and skin carcinoma was observed prior to the first hospital contact for PD, but a reduced prevalence of cancer at smoking-related sites [242].

A review of 12 studies found that melanoma occurrence was significantly higher after PD diagnosis, but not before PD diagnosis, while no significant relationship was found for non-melanoma skin cancers [243]. A history of melanoma was associated with an increased prevalence of prodromal markers of PD [244].

A UK case–control study showed an overall lower risk of developing cancer in PD vs. non-PD patients, which was strongest for smoking-related cancers (odds ratio OR 0.77/0.72). The adjusted OR for hematological malignancies was 0.32 [245]. A register-based study revealed slightly higher cancer occurrence in PD patients (hazard ratio HR 1.05), with the highest risk for those with melanoma versus non-PD (HR 1.53) [246].

A Mayo Clinic study identified 20 different cancers in PD patients; the most common cancers were skin cancer (17.3%), followed by non-melanoma skin cancer (16%), prostate cancer in men (12.8%), breast cancer in women (10.6%) and melanoma (2.4%). Compared to controls, a significantly lower frequency of non-melanoma and any kind of skin cancer was observed preceding PD diagnosis, suggesting that cancer may have a protective effect on PD risk [247]. According to a US cancer database, the age- or sex-adjusted relative risk of any melanoma was more than 7 times higher than expected from the confirmed cases in the American Academy of Dermatology skin cancer screening program [248]. A study at Duke University (2007–2020) showed 2.09 times higher odds for melanoma in PD cases compared with controls [249].

A cohort from the Israel National Cancer Registry showed an elevated prevalence of melanoma in patients with PD [250], while a population-based retrospective Israeli study found no differences in any cancer risk for the PD cohort compared to the reference population. A cohort study in southern Israel reported a positive relationship between PD and the risk of basal cell carcinoma, while weak or no associations were seen for melanoma or squamous cell carcinoma [251].

The risks of lung and colon cancers in the PD cohort were significantly lower for both sexes, while those for breast, CNS, kidney, leukemia, lymphoma, melanoma, ovarian, pancreatic, prostatic, rectal and thyroid cancer were similar in the two populations [239].

A national record-linkage study in England showed increased standardized rate ratios for six out of thirty-one cancer types, including breast, melanoma, uterus, kidney and neurological malignancies; decreased rate ratios were found for eleven cancer types, including lung and colon cancer [252]. A national cohort study in Taiwan showed a significantly higher risk of developing brain tumors in PD patients, particularly benign brain tumors, but only in the 50–64-year age group [253]. A nationwide survey in Korea showed an increased risk of melanoma in PD patients and of non-melanoma skin tumors in female PD patients over age 65 [254], while a Hungarian study reported a decreased risk of all skin tumors and an unchanged risk of melanoma among PD patients [255]. A meta-analysis of twenty-four studies (including four from China) showed a significantly increased risk of melanoma after PD diagnosis both in Europe (odds ratio/OR 1.44) and in North America (OR 1.09), while the risk of non-melanoma skin cancers was slightly higher (OR 1.20), with most of the evidence being of high quality [256]. Another review of 26 studies found a higher frequency of melanoma and non-melanoma skin cancers in PD patients than in the general population [257].

A meta-analysis of 16 studies showed a significantly higher risk of developing basal cell carcinoma in PD patients [258] and another of 63 studies (almost 18 million participants) reported a pooled relative risk of 0.82 (*p* < 0.001) for total cancer and 0.92 for non-smoking cancer, i.e., an inverse association to PD, while it was positively associated with melanoma but not other skin cancers [259]. A meta-analysis on the influence of various factors on PD and cancer revealed reduced cancer risks in PD patients (risk ratios/RR 0.77) for colon, rectal and colorectal cancer (RR 0.77) and lung cancer (RR 0.62), but increased brain cancer (RR 1.48) and melanoma (RR 1.76) risks. Compared to iPD, LRRK2 carriers had an increased general cancer risk (RR 1.26), particularly brain (RR 2.41), breast (RR 2.57), colon (RR 1.83) and hematological cancers (RR 2.05). Female PD patients had decreased cancer risks [260].

A recent cross-sectional study showed that prolonged sun exposure (*p* = 0.01) was associated with skin tumors, but no interactions were found between PD and cutaneous malignant melanoma [261].

This incomplete selection of cancer risk studies in PD, despite having deviating results, showed a general decrease in many smoking and non-smoking-related cancers in PD patients compared to controls, and a general increase in melanomas and brain tumors, and in some studies, an increase in breast, kidney and other tumors, and a general increase in LRRK2 carriers (see Table 2).

## 12. Role of Genetic Risk Factors

Earlier GWASs on PD and melanoma risk found that PD single-nucleotide polymorphisms (SNPs) identified in published GWASs do not seem to play an important role in melanoma development [264]. The GWAS summary statistics of 15 different types of cancer and the two-sample Mendelian randomization to study the causal relationship with PD found no evidence to support a causal relationship between cancers (melanoma, breast, prostate, endometrial and keratinocyte cancers) and PD, while the previously reported associations could be a result of genetic pleiotropy, shared biology or biases [265]. These data are in contradiction to many studies that have described the association of various gene mutations with an increased cancer risk in PD, such as the association of non-skin cancers with LRRK2 G2019S carriers [266,267], and PD-related genes such as LRRK2, PARKIN and αSyn increasing the risk of melanoma [268]. However, a recent study could not verify a correlation between the LRRK2 mutation and the development of malignant melanomas in either symptomatic PD patients or asymptomatic carriers, and no SNCA gene was identified in patients with a malignant melanoma history [269]. PARKIN mutations link melanoma and PD by impairing PARKIN ubiquitination activity and abolishing its tumor suppression effect [270], with the re-expression of PARKIN in melanoma cell lines resulting in reduction in cell proliferation, while the inhibition of PARKIN stimulates the proliferation of melanocytes [271]. Furthermore, the high expression of PARK6, PARK7 and PARK15 may lead to non-small-cell lung cancer [272]. The association between PD and cancer was supported by the genetic mutations of SNCA, PARK2 and PARK8, ATM, p53, PTEN and MC1R resulting in mitochondrial dysfunction, aberrant protein aggregation and cell cycle dysregulation [273]. The Parkinson gene PINK1 that regulates cell cycle progression had tumor-promoting properties [274], and like PARKIN and DJ1, acted oncogenic and, together with mitochondrial dysfunction and OS, was involved in cancer proliferation, supporting the association between PD and carcinogenesis [275]. A significant genetic correlation between melanoma and PD and overlapping gene expression across tissues was demonstrated [276]. Recent GWASs have confirmed previously reported positive genetic correlations of PD with melanoma and an additional significant correlation with prostate cancer. There was a significant inverse association between the presence of ovarian cancer and PD (odds ratio OR 0.89), while PD was positively associated with breast cancer (OR 1.08). The association between PD and ovarian cancer was mostly driven by rs183211 located in an intron of the NSF gene. These data strengthen the evidence in favor of the contribution of pleiotropic genes to the association of PD with specific cancers [277].

## 13. Linking PD and Malignancies—Possible Pathogenic Mechanisms

αSyn is associated with a variety of malignancies, including melanomas; brain tumors; hematological malignancies; colorectal, pancreatic and liver carcinomas; medulloblastomas and osteosarcomas [278,279,280,281,282,283,284,285,286,287,288]. Its expression level depends on the type of cancer. Recent studies suggest that αSyn together with other proteins are involved in various cancer-related cell processes, such as mitochondrial metabolism, autophagy or the generation of reactive oxygen species [289], that are also relevant for neurodegenerative disorders [288]. The increased expression of αSyn in malignant melanomas contributes to the increased proliferation of tumor cells [290,291,292], while the knocking out of αSyn suppresses tumor growth [293].

A reduction in αSyn expression in some tumor tissues is related to a poorer prognosis [294]. αSyn loss-of-function significantly delays melanoma onset and slows tumor growth in vivo due to the decreased DNA damage signature and increased apoptotic markers, indicating a role for αSyn in modulating the DNA response pathway [295]. Furthermore, the depletion of αSyn in melanoma cells dysregulates cellular iron metabolism leading to the accumulation of ferric iron and ferritin in cells that undergo apoptosis relative to cells expressing αSyn [293]. On the other hand, high levels of αSyn expression in melanoma cells help them to evade immune responses against SNCA cells by inhibiting the secretion of immune activating factors [296].

Patients with PD and melanoma have increased staining for αSyn in their skin, suggesting that neurons and melanocytes, both derived from neuroectodermal cells, may share protein synthesis and regulation pathways that become dysfunctional in both disorders [292]. Proteinaceous αSyn amyloid fibrils are present in intracellular inclusions of PD brains and have also been detected in cultured melanoma cells and tissues derived from patients with melanomas, where an inverse correlation exists between αSyn expression and pigmentation. Although this has led to the prevailing hypothesis that αSyn inhibits enzymes involved in melanin biosynthesis, an alternative hypothesis suggests that αSyn interacts with and modulates the aggregation of Pmel17, a functional amyloid that serves as a scaffold for melanin biosynthesis, thus defining a molecular link between PD and melanoma [297,298]. This appears plausible, because αSyn amyloids produce deleterious gain-of-function effects and may cause mitochondrial dysfunction and protein degradation failure [299,300].

Although clinical and epidemiological associations between PD and cancer, in particular melanoma, are well established, the causal relationships between the two disorders are not fully explored. In particular, the genetic, cellular and molecular pathways linking these diseases are poorly understood. Accumulating data point to the roles of genetic traits, synucleins, neurotrophic factors, DNA damage and other shared molecular factors that are involved in the association between PD and cancer (for a review, see [301]).

**Figure 1:**
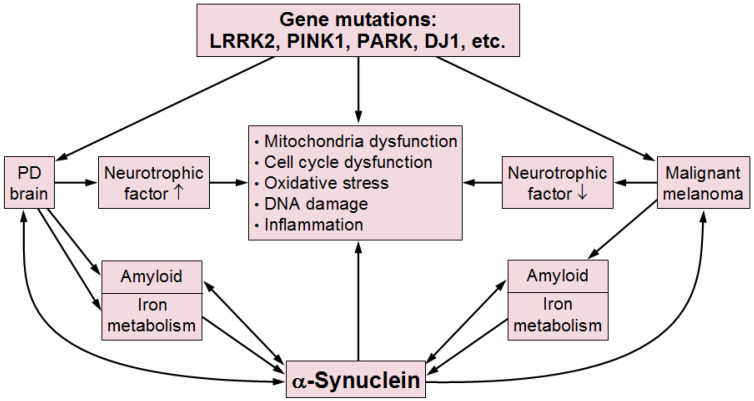
Some pathogenic factors implicated in PD and cancer. Arrows indicate causal influences between the factors. PD: Parkinson disease.

In conclusion, common pathogenic mechanisms underlie both PD and cancer, encompassing immune signaling, DNA damage, mitochondrial dysfunction, metabolic alterations, cell cycle defects, inflammation and the dysregulation of cellular iron metabolism, mechanisms that, together with gene expression modulation, are implicated in both malignant processes and PD. Among the multitude of pathogenetic factors, only some share genetic risk factors between PD and cancer, and of the specific molecular mechanisms underlying synuclein’s role, only some are depicted in Figure 1.

## 14. Conclusions and Outlook

Coexisting neuropathological comorbidities have been repeatedly reported to be common in the great majority of subjects with PD as well as in other neurodegenerative diseases [4,5,6]. Most of them are more common than (or comparable to) those in clinically normal elderly people and may have an impact on the evolution and symptomatology of the basic disease. It has been suggested that many of them are likely present even before the development of the typical signs and symptoms of PD. The coexistence of PD, AD, cerebrovascular pathology and several malignancies, in particular melanomas, as well as of many autoimmune diseases and diabetes mellitus, is extremely common, whereas comorbidity with MS and other neurodegenerative disorders, e.g., PSP and ALS, is less frequent, while concomitant Pick’s disease has not been found [15]. In contrast, the FDP-43 pathology, AGD and ARTAG, which are frequent phenomena in old-aged subjects, are also common in PD brains. The prevalence of cerebrovascular lesions in PD is higher than in age-matched controls [53,56,57], while the association with cardiovascular risk factors is decreased, probably related to αSyn-associated hypotension [15,302].

For some of these age-related co-pathologies, it is difficult to disentangle their individual contribution to disease progression and cognitive decline [66,303], but, in general, a combination of various co-pathologies is responsible for the development and progression of cognitive and other clinical deficits in PD [9,66].

**Figure 2:**
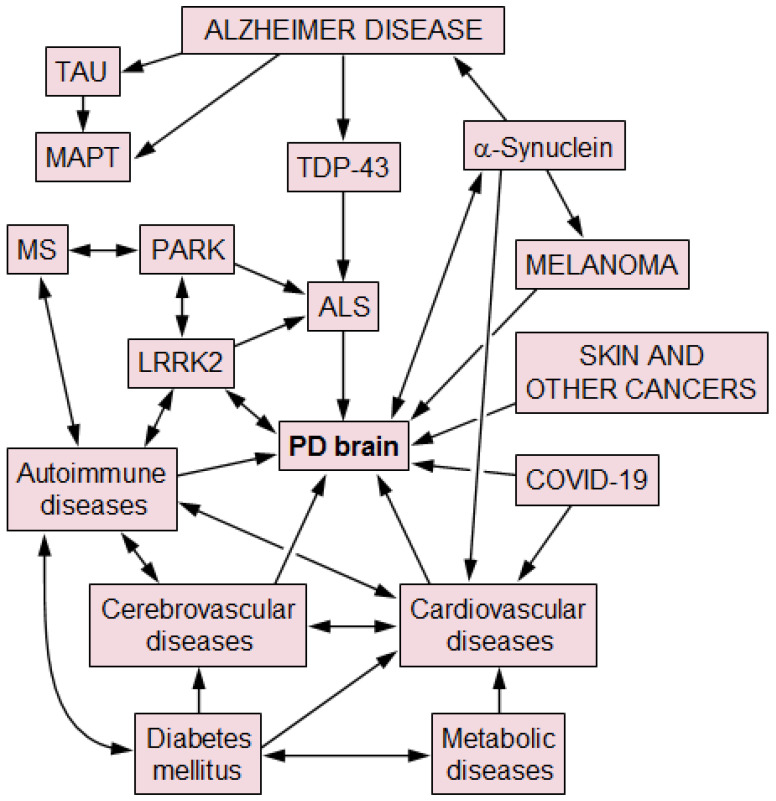
Hypothetical relationship between Parkinson disease and other pathologies. Arrows indicate causal influences between pathologies. PD: Parkinson disease; MS: multiple sclerosis; and ALS: amyotrophic lateral sclerosis.

The frequent concurrence of neurodegenerative disorders is suggested to be due to complex interaction of αSyn, Aβ, tau and other pathological proteins like TDP-43 that clearly concur with aging [20,304], most probably affecting multiple separate pathways or interrelated molecular mechanisms that result in the complex interplay of disease progression and cognitive decline in PD (see Figure 2). Many of these mechanisms may be influenced by genetic variants, especially those involving protein transport/clearance, autophagy and lysosomal and mitochondrial function [260,305], while the influences of environmental and other factors are poorly known.

Since co-pathologies have a considerable impact on the course and outcome of PD and, therefore, may involve the outcome and quality of life of the patients, it is necessary to assess at an early stage the probability that these comorbidities will affect persons with PD and to clarify their impact on the disease process and its complications. A deeper understanding of the interactions of the multiple pathogenic factors is warranted to contribute to a possible inhibition and/or reduction in the progression of at least some of these complicating comorbidities.

## Figures and Tables

**Table 2:** Parkinson disease and cancer risk/incidence.

Reduced Risk/Incidence Smoking-Related/Non- Smoking	Increased Risk/Incidence	No Differences vs. Controls	Study Type	References
Lung, colorectal, bladder, prostate cancer	Malignant melanoma		Cohort analysis	[240]
Smoking-related cancer	Melanoma, skin carcinoma (early PD)		Danish Hospital Register	[242]
	Hematologic malignancies		UK case–control	[245]
	Melanoma 7 times higher		US cancer database	[248]
All cancers 31% ↓; after excluding skin tumors, 27% ↓	Skin tumors		29 studies (107,598 PD patients)	[241]
Cancer	Melanoma, non-melanoma skin cancer, breast cancer		26 studies	[257]
	Melanoma		Israel National Cancer Registry	[250]
	Melanoma + PD vs. non-PD OR = 2.11 Melanoma after PD diagnosis not significantly increased	Non-melanoma skin tumors	21 studies	[243]
	Melanoma 40% ↑ (before and after PD diagnosis)		Register-based	[246]
Overall cancer	Melanoma, non-melanoma skin cancer, breast cancer	Cancer (early PD)	Danish register	[238]
	Melanoma (prodromal PD)		Prospective study	[244]
11 cancers (lung, colon)	Six cancers (breast, uterus, melanoma, kidney, neurological)		UK nationwide	[252]
Skin cancer (non-melanoma) preceding PD	Melanoma, breast and prostate cancer		Mayo Clinic Register	[247]
Cancer	Melanoma, breast carcinoma		Minnesota	[262]
Lung, colon cancer	Brain tumors		Taiwan cohort	[253]
	Basal cell carcinoma	Melanoma	South Israel cohort	[251]
Lung, colon cancer		Most malignancies	Population-based cohort	[239]
	Melanoma, non-melanoma skin tumors (females over age 65)		Korea nationwide	[254]
	Melanoma (after PD diagnosis), in Europe and North America: non-melanoma skin cancer slightly ↑		24 studies	[256]
	Basal cell carcinoma ↑↑		16 studies	[258]
	Total cancers, melanoma	Other skin cancers	63 studies (*n* = 18 million)	[259]
Lung, colon, rectal, colorectal cancer	Brain, breast, colon, hematological, melanoma LRRK2-G2019S carriers		14 studies	[260]
Pancreatic, colorectal cancer	Liver cancer		Global Health Observatory database	[263]
	Melanoma 2 times ↑		Duke University study	[249]
All tumors		Melanoma	Hungary	[255]
		Melanoma	141 PD patients	[261]

PD: Parkinson disease; OR: odds ratio.

## Supplementary Materials

The following supporting information can be downloaded at: https://www.mdpi.com/article/10.3390/ijms26072942/s1.

## Abbreviations

AD	Alzheimer disease
ADNC	Alzheimer disease-related pathology
AiDs	autoimmune disorders
ALS	amyotrophic lateral sclerosis
ARTAG	aging-related tau astrogliopathy
Aβ	β-amyloid
αSyn	α-synuclein
CAA	cerebral amyloid angiopathy
CD	Crohn’s disease
CJD	Creutzfeldt–Jakob disease
CSVD	cerebral small vessel disease
CVP	cerebrovascular pathology
DLB	dementia with Lewy bodies
FD	Fabry disease
GWAS	genome-wide association studies
IBD	inflammatory bowel disease
LATE	limbic-predominant age-related TDP-43 encephalopathy
LBs	Lewy bodies
MG	myasthenia gravis
Mr	Mendelian randomization
MS	multiple sclerosis
OS	oxidative stress
PD	Parkinson disease
pSS	primary Sjögren’s syndrome
RA	rheumatoid arthritis
RLS	restless leg syndrome
SN	substantia nigra
SN	substantia nigra
SS	Sjögren’s syndrome
T2DM	type 2 diabetes mellitus
WM	white matter
WM	white matter
