# Supplementary material for: Concomitant Pathologies and Their Impact on Parkinson Disease: A Narrative Overview of Current Evidence
Source: Int J Mol Sci. 2025 Mar 24;26(7):2942. doi: 10.3390/ijms26072942 (PMC11988849; doi:10.3390/ijms26072942)
Supplement: Supplementary file 1 [file ijms-26-02942-s001.zip › ijms-3525803-supplementary.pdf]

Review - Int. J. Mol. Sci.
Special Issue "Emerging Concepts of Neurodegenerative Research"

Concomitant pathologies and their impact on Parkinson disease. A narrative overview of current evidence

Kurt A. Jellinger
Institute of Clinical Neurobiology, Vienna, Austria

Supplementary Material: Search strategy

A comprehensive literature research was conducted through the databases PubMed, Google Scholar, Web of Science, and Cochran Library, to identify studies indexed on these platforms between 2000 and January 2025. Since this was not a systematic literature review, the PRISMA method was not followed.

1. Inclusion criteria: Search was performed according to the following MeSH (Medical Subject Headings) keywords: Alzheimer disease, AD-related lesions, amyotrophic lateral sclerosis, arteriolosclerosis, atherosclerosis, argyrophilic grains, arthritis, autoimmune diseases, cardiovascular disease, cerebrovascular lesions, cerebral amyloid angiopathy (CAA), co-morbidities, co-pathologies COVID-19, diabetes mellitus type 1 and 2, endocrine disturbances, fragile X syndrome, hippocampal sclerosis, inflammatory bowel disease, lacunes, metabolic syndrome, microinfarcts, musculoskeletal disorders, myasthenia gravis, restless leg syndrome, rheumatoid disorders, Sjögren's syndrome, TDP-43 pathology. The results were further screened by title, and only articles containing the above subjects and those in English or with English abstract were included.

2. Exclusion criteria: Case reports, conference abstracts and reports, commentaries, letters, duplicate studies, non-English articles without English abstracts and studies focusing on neuropsychiatric co-morbidities and symptoms including depression, psychoses, hallucinations. Since the search was limited to Parkinson's disease, atypical parkinsonian syndromes were not considered and should deserve a special review. The review does not consider viral parkinsonism.

Subsequently, the selected articles (after de-duplication more than 1,000) were extracted and reviewed manually by the author using the abstract and, if available, the full article.
